# Validation of numerical simulation methods in aortic arch using 4D Flow MRI

**DOI:** 10.1007/s00380-017-0979-2

**Published:** 2017-04-25

**Authors:** Shohei Miyazaki, Keiichi Itatani, Toyoki Furusawa, Teruyasu Nishino, Masataka Sugiyama, Yasuo Takehara, Satoshi Yasukochi

**Affiliations:** 1 Cardio Flow Design, Inc., Chiyoda, Tokyo Japan; 2 Department of Cardiovascular Surgery, Kyoto Prefectural University of Medicine, 1-5 Hangi-cho, Shimogamo, Sakyo-ku, Kyoto, 606-8522 Japan; 3 NEC Solution Innovators, Ltd., Koutou, Tokyo Japan; 4 Department of Radiology, Hamamatsu University School of Medicine, Hamamatsu, Shizuoka Japan; 5 Department of Pediatric Cardiology, Nagano Children’s Hospital, Azumino, Nagano Japan

**Keywords:** Phase-contrast MRI, Computational fluid dynamics, Wall shear stress, Flow energy loss

## Abstract

Computational fluid dynamics (CFD) are the gold standard in studying blood flow dynamics. However, CFD results are dependent on the boundary conditions and the computation model. The purpose of this study was to validate CFD methods using comparison with actual measurements of the blood flow vector obtained with four-dimensional (4D) flow magnetic resonance imaging (MRI). 4D Flow MRI was performed on a healthy adult and a child with double-aortic arch. The aortic lumen was segmented to visualize the blood flow. The CFD analyses were performed for the same geometries based on three turbulent models: laminar, large eddy simulation (LES), and the renormalization group k–ε model (RNG k–ε). The flow-velocity vector components, namely the wall shear stress (WSS) and flow energy loss (EL), of the MRI and CFD results were compared. The flow rate of the MRI results was underestimated in small vessels, including the neck vessels. Spiral flow in the ascending aorta caused by the left ventricular twist was observed by MRI. Secondary flow distal to the aortic arch was well realized in both CFD and MRI. The average correlation coefficients of the velocity vector components of MRI and CFD for the child were the highest for the RNG k–ε model (0.530 in ascending aorta, 0.768 in the aortic arch, 0.584 in the descending aorta). The WSS and EL values of MRI were less than half of those of CFD, but the WSS distribution patterns were quite similar. The WSS and EL estimates were higher in RNG k–ε and LES than in the laminar model because of eddy viscosity. The CFD computation realized accurate flow distal to the aortic arch, and the WSS distribution was well simulated compared to actual measurement using 4D Flow MRI. However, the helical flow was not simulated in the ascending aorta. The accuracy was enhanced by using the turbulence model, and the RNG k–ε model showed the highest correlation with 4D Flow MRI.

## Introduction

The flow visualization methods used with recent imaging technologies have been applied to the circulatory system to reveal the pathophysiology of cardiovascular diseases. Computational fluid dynamics (CFD) are considered a gold standard method of blood flow visualization and are often used for analyzing blood flow in cases of aortic disease to evaluate the wall shear stress (WSS) [1–3] or in cases of congenital heart disease to evaluate the flow energy loss (EL) [4, 5]. Compared to in vivo measurements, including echocardiography or phase contrast MRI, CFD has various advantages. CFD allows testing of the effects of isolated factors, allowing blood flow evaluation without statistical study. Moreover, CFD also enables virtual surgery by modifying the vessel geometry into the intended post-operative geometry. The high spatial and temporal resolution in CFD enables precise evaluation of blood flow in small vessels such as the coronary artery. In addition, the spatial and temporal resolutions can be increased as far as the computational costs permit. The spatial resolution, which is defined by the computing mesh, can be partially increased, for example, in the near-wall region or the stenotic arterial region, to obtain refined results including WSS and EL [6]. However, the CFD computation is based on computational models such as the turbulence model that must be appropriate to the computing subject in order to compute the actual flow, which is the most difficult part when using CFD in the cardiovascular system.

Accurately computing the turbulence is important for computing both EL and WSS, and a better understanding of the flow and flow turbulence in the aortic arch leads to more accurate computation of these parameters. Many studies have been conducted numerically or experimentally for understanding the turbulence in vasculature. For example, Jahangiri et al. used fluid structure interaction for evaluating the turbulence in a stenosed artery. Using the standard k–ε model, renormalization group (RNG) k–ε model, and laminar scheme for computing the flow in a coronary artery with 80% stenosis, they reported that the length of oscillatory region, which is used for describing how the disease is spread, decreased by using the turbulence model [7]. Failure to consider the turbulent flow behaviour can cause a large numerical error. Kousera et al. reported stability of aortic flow [8]. Using the Reynolds-averaged Navier–Stokes-based shear stress transport transitional model to compute various Reynolds numbers ranging from 4000 to 10,000 and Womersley parameters ranging from 17 to 26, they concluded that the model is capable of capturing the correct flow state by comparing the results to experimentally acquired in vivo flow measurements using a catheter-mounted hot-film probe.

However, there are few studies about turbulence in the aortic arch based on in vivo velocity vector measurements, and there are no studies about the effect of turbulence models on the aortic arch flow that is formed with a three-dimensionally (3D) twisted aortic arch and vessel wall irregularity. Further, there are no indications regarding the use of turbulence models.

Recently, four-dimensional (4D) flow magnetic resonance imaging (MRI) has become a powerful tool for analyzing blood flow in vivo. Even though the spatial and temporal resolution of 4D Flow MRI is insufficient for calculating the EL and WSS [3, 9], it is the only method for measuring the 3D distribution of the blood vector field in vivo.

The aim of this study was to evaluate the use of turbulence models in the CFD of the aortic flow and to validate the effects of such models on the flow in the aortic arch through comparison with 4D Flow MRI.

## Materials and methods

Both 4D Flow MRI and CFD were used to visualize blood flow and calculate WSS and flow EL in a healthy adult and a child with double aortic arch (DAA). The 3D velocity distribution was systemically validated, and WSS and EL were compared between 4D Flow MRI and CFD. This study was specifically approved by local institutional review boards (Kitasato University Medical Ethics Organization B13-140; The Ethics Committee of the Hamamatsu University School of Medicine 25–246, 25–335, 22–45; and The Ethics Committee of Nagano Children’s Hospital). Written informed consent was obtained from all participants and from the guardian on behalf of the child participant.

### Patients and MRI data acquisition

Time-resolved 3D blood flow imaging was applied to a healthy female adult volunteer (49 years old) without any known cardiovascular disease and to a male child patient with DAA (8 years old). Cardiac-gated 4D Flow MRI sequences were acquired with a breath hold using the parameters described in Table 1. In the healthy adult, steady-state free precession (SSFP) imaging was applied to the same position with the same spatial resolution used for segmentation.

**Table 1:** 4D Flow MRI acquisition parameters

	Healthy adult	Child with DAA
MRI machine	GE Signa HDxt 1.5T	Philips Multiva 1.5T
TR (ms)	4.59	4.97
TE (ms)	2.12	2.41
Flip angle (degree)	15	10
FOV (mm)	320	200
Slice thickness (mm)	2	1
View per cardiac phase	20	12
Temporal resolution (ms)	49.2	41.7
Image matrix size	256 × 256 × 60	224 × 224 × 50
Voxel size (m^3^)	1.25 × 1.25 × 2.00	0.885 × 0.885 × 1.00
Readout direction	A/P	R/L

### Data processing and geometry reconstruction

Only the peak systolic phase was targeted for validation. To avoid the position gap between 4D Flow MRI and CFD, the aortic lumen was segmented in the systolic phase. The segmented lumen was used for both 4D Flow MRI analysis and CFD analysis.

In-house software coded in C++/CLI was used to analyze and segment the acquired raw data. Systolic peak images were used for the segmentation and numerical validation because velocity data were used for the voxel-by-voxel boundary definition as described below. In the segmentation method, the aorta volume was initially segmented coarsely using a threshold that covered the full volume of the aorta, and the 3D region growing method was then applied.

**Fig. 1:**
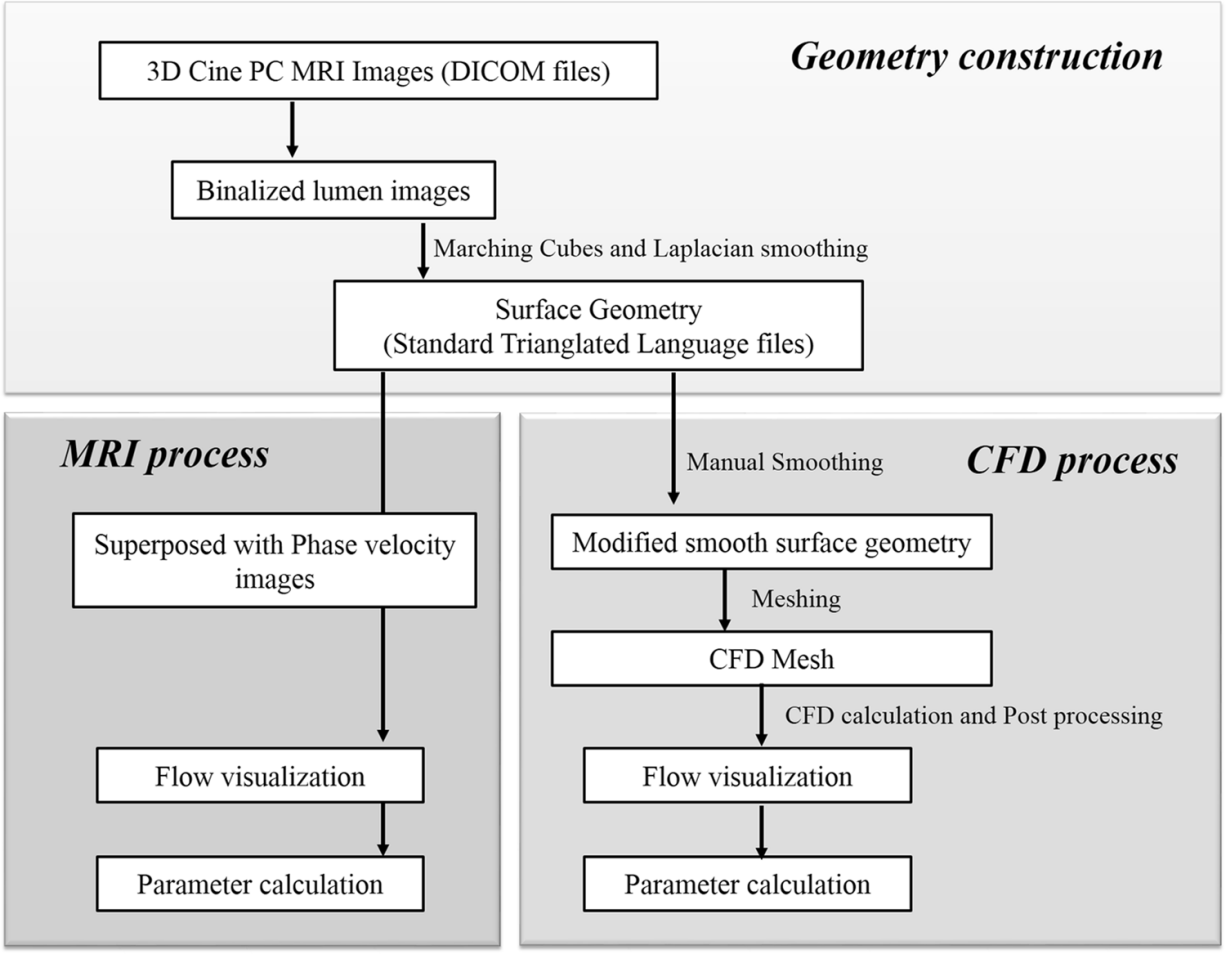
The flow visualization process using 4D Flow MRI and CFD

The contrast of the magnitude images between the inner and outer cavities of the vessels was sufficiently high to precisely define the boundary surface; therefore, we used the phase velocity images to define the boundary surface. After this process, the boundary voxels of the segmented images were manually modified one-by-one through comparison with the velocity images (Fig. 1). To produce a sufficiently smooth surface, the marching cubes method was applied to the segmented volume followed by Laplacian smoothing.

The streamline, flow rate, and flow EL were calculated from the volume data, and WSS was calculated from the smoothed surface data and the near-wall velocity data. Open-source visualization software, ParaView 3.8.2 (Sandia National Labs, Kitware Inc., Los Alamos National Labs), was used to visualize the streamline and WSS and to calculate the flow rate.

The following method was used to calculate WSS on each surface mesh: the velocity on the vessel surface was assumed to be zero; the velocity at a half voxel inner site along the normal direction from the center of the surface mesh was calculated by linear interpolation; and the projection of the velocity vector parallel to the surface mesh was calculated to define the wall shear vector. The WSS in each mesh was calculated according to

1 $${\text{WSS}} = \mu\frac{{\left| {\overrightarrow {\text{WSS}} } \right|}}{{\left( {{\raise0.7ex\hbox{$1$} \!\mathord{\left/ {\vphantom {1 2}}\right.\kern-0pt} \!\lower0.7ex\hbox{$2$}}|\vec{n}|} \right)}} ,$$

where $\vec{n}$ is the unit normal vector of each mesh. Furthermore, EL was calculated according to

2 $${\text{EL}} = \int {(\mu )\sum\limits_{i,j} {\frac{1}{2}\left( {\frac{{{\partial}u_{i} }}{{{\partial}x_{j} }} + \frac{{{\partial}u_{j} }}{{{\partial}x_{i} }}} \right)^{2} \text{d}V} } ,$$

where *μ* is the viscosity of the blood (*μ* = 0.004 Pa·s) [10].

### CFD method

The CFD method was based on previous studies [6, 11]. The CFD mesh was generated on the smooth surface geometry of the inner vessel cavity that was generated in the 4D Flow MRI postprocessing. Additional smoothing was manually applied, and coronary arteries that were not clearly resolved by MRI were reformed using 3D graphic software, Blender 2.66 (Blender Foundation, Amsterdam, Netherlands), before generating the computational mesh.

A commercial CFD mesh generator, ANSYS-ICEM 14.5 (ANSYS Japan, Tokyo, Japan), was used for the computational grid creation. The inner volumes of the aorta were meshed by 700,000 tetrahedral cells and 380,000 prism cells with 330,000 nodes for the healthy adult and 520,000 tetrahedral cells and 440,000 prism cells with 330,000 nodes for the child with DAA. Prism cells, which had five layers with a 30-µm-thick outermost layer, were applied to the near-wall region. Inlet boundary meshes were extended by five times the length of their diameters to develop the velocity profile of ejected flow, and the outlet boundary meshes were extended 50 times the length of their diameter for sufficient pressure recovery and to stabilize the flow split over multiple outlets.

The following inlet boundary conditions were used: the flow rate at the aortic valve was measured using 4D Flow MRI; the measured velocity distribution was integrated and calculated as the volume flow and spline interpolated along the time axis; the volume flow was converted into velocity at the CFD inlet boundary; and a flat velocity profile was given to the top of the extruded boundary. The outlet boundary condition was a pressure condition of the reflection wave from the peripheral arteries [12]. Rigid wall boundary conditions were used.

The commercial CFD solver, ANSYS Fluent 14.5 (ANSYS Japan, Tokyo, Japan), was used for computation. The following solver settings were used: the pressure implicit with splitting of operator (PISO) method was used as the pressure–velocity coupling method, the second-order backward Euler method was used as the discretization method, and the time step size was 5.0 × 10^−5^ s. The residual errors for the convergence criteria were set to <1.0 × 10^−5^. Three numerical turbulence calculation methods were applied: a laminar scheme, a large eddy simulation (LES) using the Smagorinsky–Lilly model, and the RNG k–ε model. The commercial CFD postprocessing software, ANSYS-CFX-Post 14.5 (ANSYS Japan, Tokyo, Japan), was used to analyze and visualize the numerical CFD results. The flow EL was calculated according to

3 $${\text{EL}} = \int {\left( {\mu + \mu_{t} } \right)} \sum\limits_{i,j} {\frac{1}{2}\left( {\frac{{{\partial}u_{i} }}{{{\partial}x_{j} }} + \frac{{{\partial}u_{j} }}{{{\partial}x_{i} }}} \right)^{2} {\text{d}}V}$$

4 $$\mu_{t} = 0\,\left( {\text{Laminar scheme}} \right)$$

5 $$\mu_{t} = \frac{{{\text{C}}_{\mu }\uprho{\text{k}}^{2} }}{\varepsilon } \left( {{\text{RNG k}} - \varepsilon } \right)$$

6 $$\mu_{t} = \left( {{\text{C}}_{\text{s}} \Delta_{g} } \right)^{2} \sqrt {2S_{ij} S_{ij} } \left( {\text{LES}} \right),$$

where *μ* is the viscosity of the blood (*μ* = 0.004 Pa·s), *μ*_t_ is the eddy viscosity of the turbulence model, *C*_*μ*_ and *C*_*s*_ are constants (*C*_*μ*_ = 0.0845, C_*s*_ = 0.1), Δ is the local grid size, and *S* is the velocity strain tensor [11].

### Numerical comparison of CFD and MRI

The velocity distribution and WSS of CFD and 4D Flow MRI were compared in the systolic peak phase in order to avoid the geometry mismatch between the methods. The velocity distribution was compared in three parts: the ascending aorta (AAo), aortic arch, and descending aorta (DAo). To compare velocities in the same coordinates, the CFD velocity distribution was obtained on the MRI structure grid coordinates using linear interpolation of the CFD mesh. These coordinate transforms with interpolation were performed using in-house code with Matlab 2012b (MathWorks Japan, Tokyo, Japan).

## Results

### Velocity verification

**Fig. 2:**
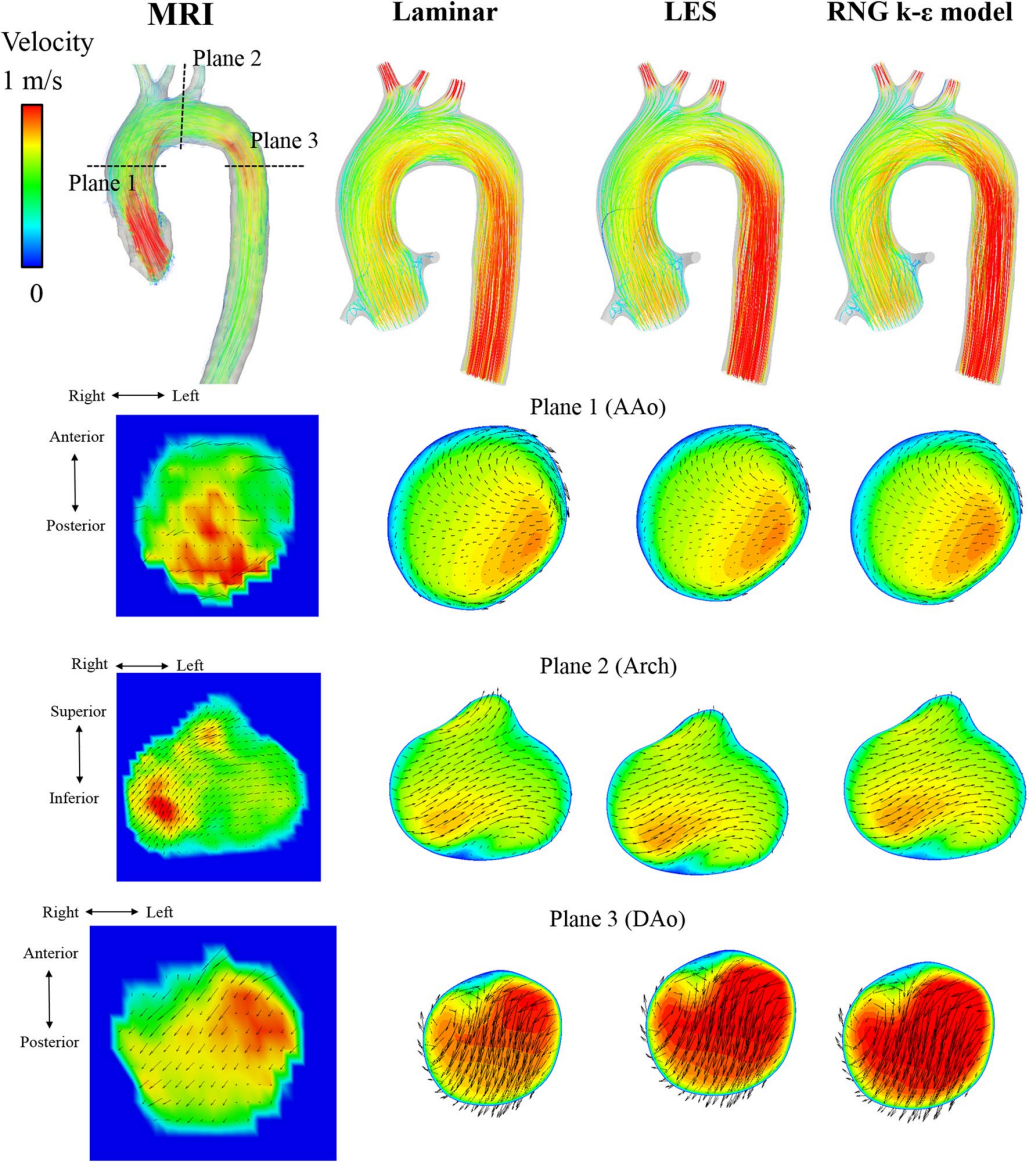
Flow in the healthy adult. Streamline and in-plane velocity distributions of the systolic peak are compared among 3D Cine PC MRI and three CFD computations

**Fig. 3:**
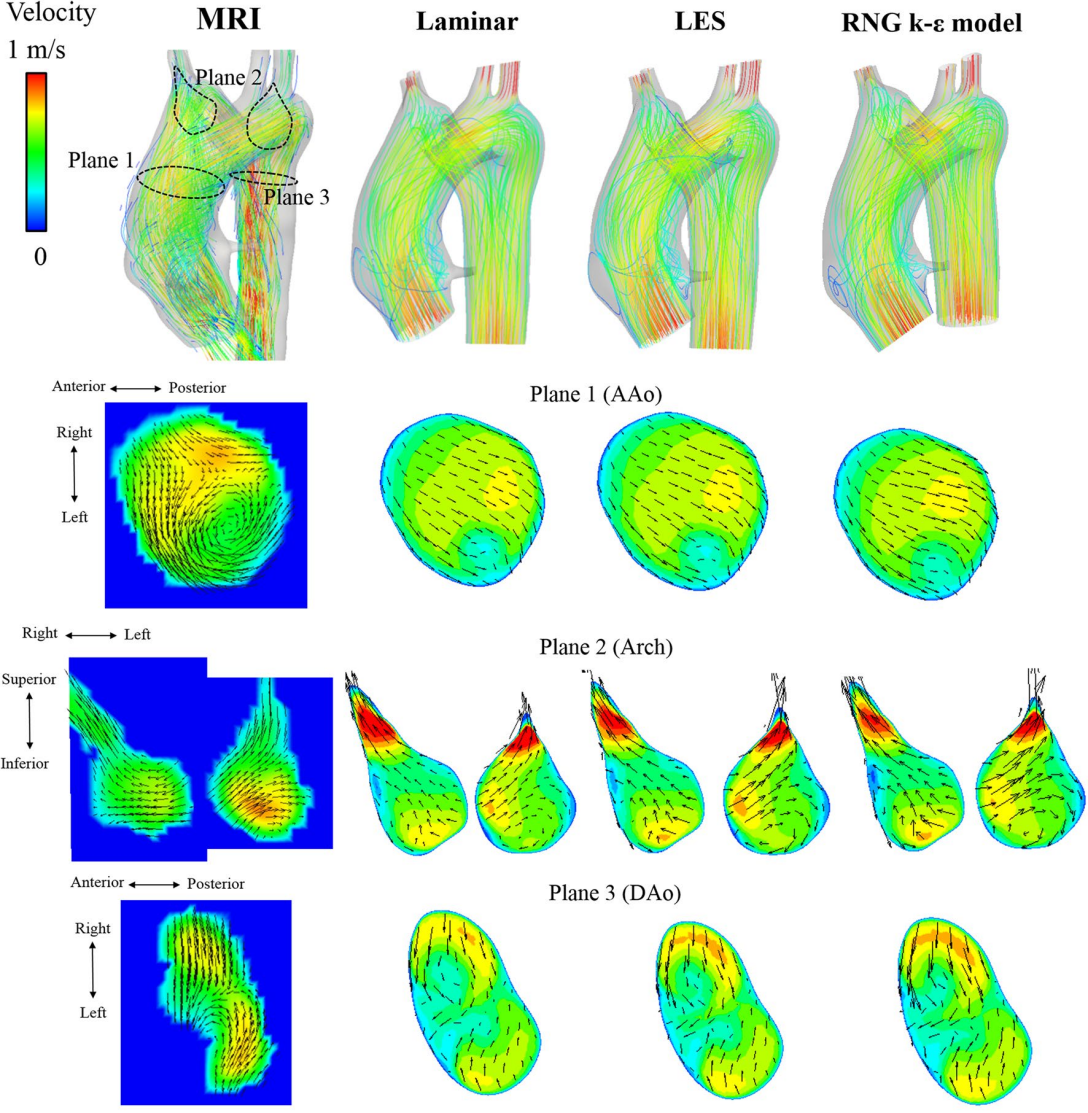
Flow in the child with DAA. Streamline and in-plane velocity distributions of the systolic peak are compared among 4D Flow MRI and three CFD computations. In the ascending aorta, helical flow is observed in 4D Flow MRI, whereas straight flow is computed in CFD

**Fig. 4:**
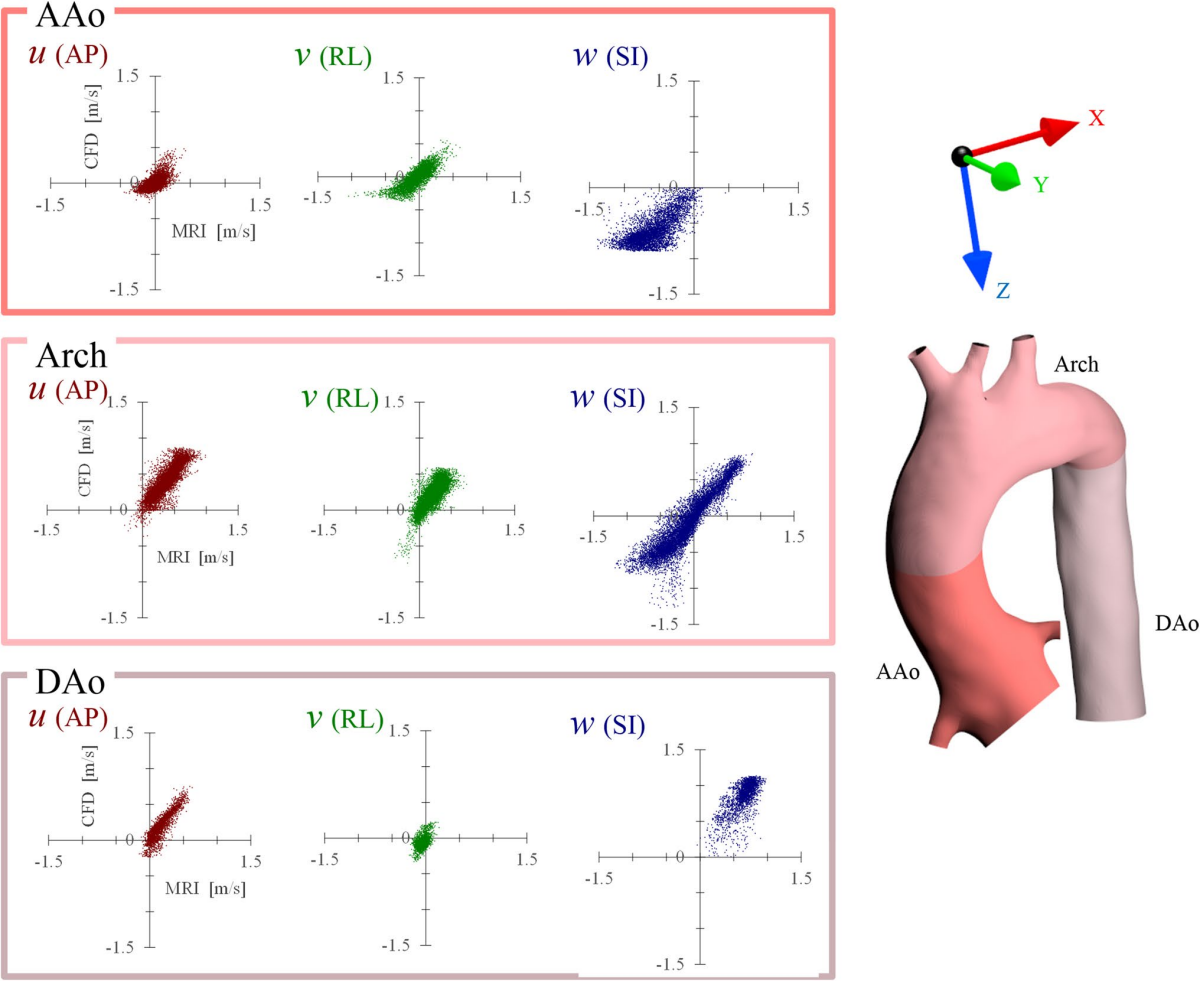
Correlation between 4D Flow MRI and CFD (RNG k–ε) in the healthy adult. Flow vector components of anterior–posterior (AP) direction, *right*–*left* (RL) direction, and superior–inferior (SI) direction are compared in the ascending aorta (AAo), aortic arch (Arch), and descending aorta (DAo). The components in each part are well correlated

**Fig. 5:**
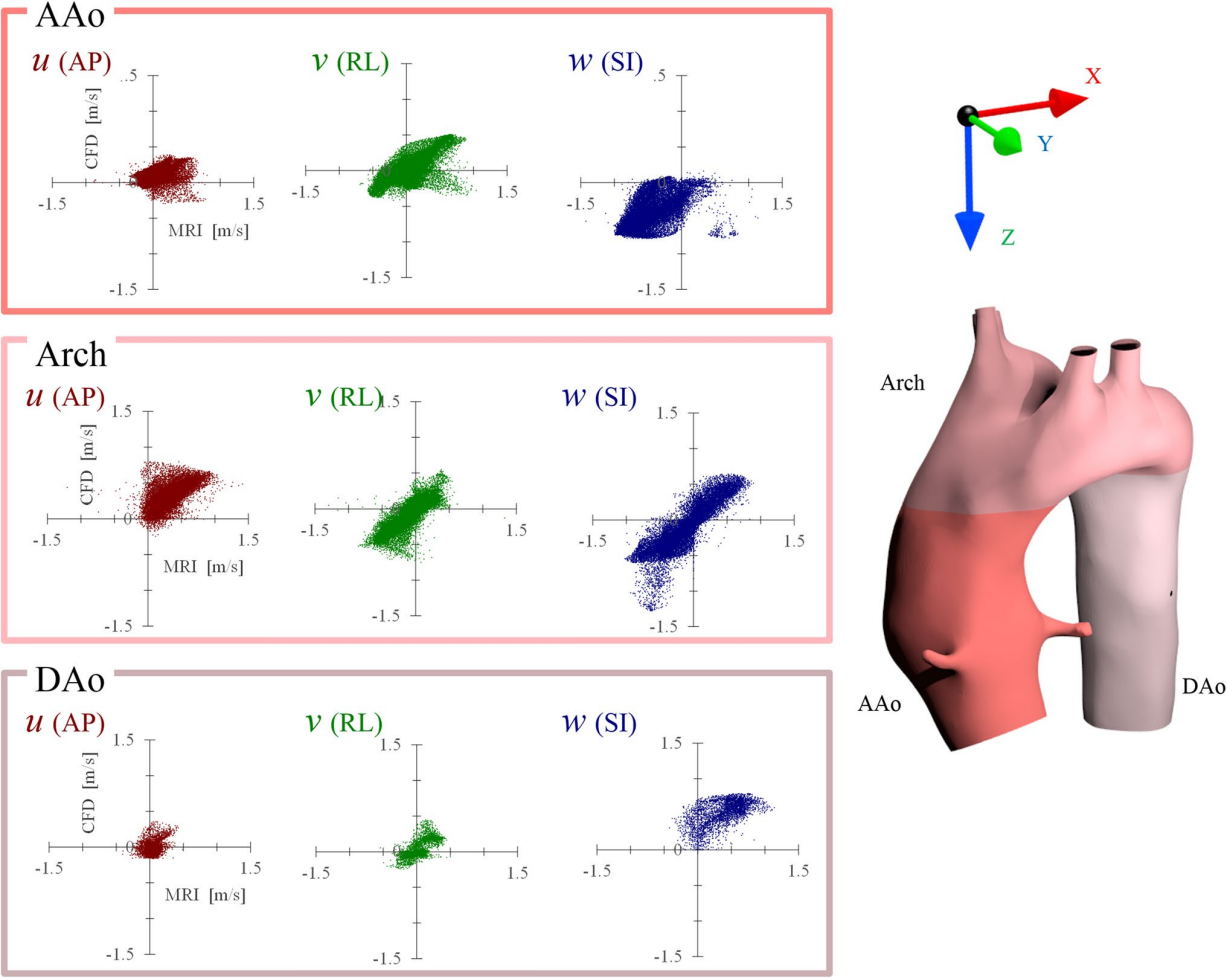
Correlation between 4D Flow MRI and CFD (RNG k–ε) in the child with DAA. In the child, the flow vector components of the primary flow direction (*w* in AAo, *u* and *w* in the arch, *w* in DAo) are well correlated, but the secondary flow directional components show comparatively low correlation in AAo

The streamline and velocity on the three typical planes of 4D Flow MRI and CFD are shown in Fig. 2 for the healthy adult and in Fig. 3 for the child. The vector on the plane is the in-plane velocity vector, and the contour shows the velocity magnitude. Table 2 shows the Pearson product-moment correlation coefficient and area-averaged error of the velocity-vector components between MRI and the three CFD models for each part, and Figs. 4 and 5 show the correlation diagrams for the adult and child, respectively. All of the correlations were sufficiently high and statistically significant (*P* < 0.00000001), irrespective of the turbulence models, velocity direction, or parts of the aortic arch. In the child, helical flow was clearly observed by MRI, whereas comparatively straightforward flow was realized by CFD. Thus, the correlation of the velocity components in the secondary flow was lower than that in other parts. On the other hand, the CFD flows in the middle of the aortic arch and the distal arch were very similar to those of MRI, and the correlations were higher than those in AAo.

**Table 2:** Correlation coefficients and errors of CFD with 4D Flow MRI

	Number of sampling points	Laminar		
		*u* (AP)	*v* (RL)	*w* (SI)
(a) Laminar scheme				
Healthy adult				
Correlation coefficient				
AAo	4643	0.640	0.795	0.654
Arch	9609	0.848	0.786	0.903
DAo	2507	0.905	0.656	0.822
Error (m/s)				
AAo	4643	0.068 ± 0.054	0.087 ± 0.073	0.158 ± 0.128
Arch	9609	0.077 ± 0.071	0.077 ± 0.069	0.111 ± 0.125
DAo	2507	0.071 ± 0.052	0.073 ± 0.049	0.142 ± 0.087
Child with DAA				
Correlation coefficient				
AAo	19,863	0.300	0.671	0.560
Arch	16,552	0.596	0.820	0.855
DAo	10,894	0.434	0.672	0.536
Error (m/s)				
AAo	19,863	0.129 ± 0.111	0.150 ± 0.142	0.210 ± 0.172
Arch	16,552	0.128 ± 0.111	0.110 ± 0.092	0.147 ± 0.125
DAo	10,894	0.093 ± 0.070	0.111 ± 0.084	0.185 ± 0.149

	LES		
	*u* (AP)	*v* (RL)	*w* (SI)
(b) LES			
Healthy adult			
Correlation coefficient			
AAo	0.642	0.800	0.659
Arch	0.872	0.804	0.922
DAo	0.909	0.664	0.816
Error (m/s)			
AAo	0.068 ± 0.054	0.086 ± 0.072	0.157 ± 0.128
Arch	0.086 ± 0.073	0.081 ± 0.065	0.112 ± 0.103
DAo	0.087 ± 0.059	0.079 ± 0.053	0.184 ± 0.098
Child with DAA			
Correlation coefficient			
AAo	0.300	0.673	0.565
Arch	0.599	0.821	0.863
DAo	0.439	0.678	0.549
Error (m/s)			
AAo	0.130 ± 0.111	0.150 ± 0.142	0.209 ± 0.172
Arch	0.127 ± 0.112	0.109 ± 0.092	0.144 ± 0.121
DAo	0.094 ± 0.070	0.111 ± 0.085	0.183 ± 0.150

	RNG k-ε		
	*u* (AP)	*v* (RL)	*w* (SI)
(c) RNG k–ε			
Healthy adult			
Correlation coefficient			
AAo	0.644	0.812	0.674
Arch	0.865	0.795	0.918
DAo	0.914	0.662	0.844
Error (m/s)			
AAo	0.068 ± 0.054	0.084 ± 0.070	0.153 ± 0.126
Arch	0.083 ± 0.073	0.082 ± 0.067	0.112 ± 0.107
DAo	0.088 ± 0.059	0.077 ± 0.051	0.181 ± 0.093
Child with DAA			
Correlation coefficient			
AAo	0.304	0.694	0.592
Arch	0.604	0.826	0.873
DAo	0.482	0.694	0.576
Error (m/s)			
AAo	0.127 ± 0.109	0.150 ± 0.134	0.202 ± 0.159
Arch	0.125 ± 0.111	0.107 ± 0.090	0.139 ± 0.115
DAo	0.090 ± 0.067	0.108 ± 0.082	0.179 ± 0.149

The velocity in the neck vessels obtained by MRI was low. Table 3 shows the mass flow rates in AAo, the neck vessels, and DAo. In MRI, the flow conservation law was not satisfied between the inlet and outlets, and it was believed that the flow rate in the neck vessels was highly underestimated and that in DAo was mildly underestimated.

**Table 3:** Flow rate at the AAo and the outlets

	MRI	Laminar	LES	RNG k–ε
Healthy adult (kg/s)				
AAo	0.331	0.342	0.342	0.342
Upper body (neck branches)	0.038	0.102	0.0808	0.0757
Lower body (DAo)	0.187	0.237	0.258	0.263
Child with DAA (kg/s)				
AAo	0.219	0.215	0.215	0.215
Upper body (neck branches)	0.0282	0.0809	0.0754	0.0713
Lower body (DAo)	0.118	0.134	0.139	0.143

### WSS and EL

**Fig. 6:**
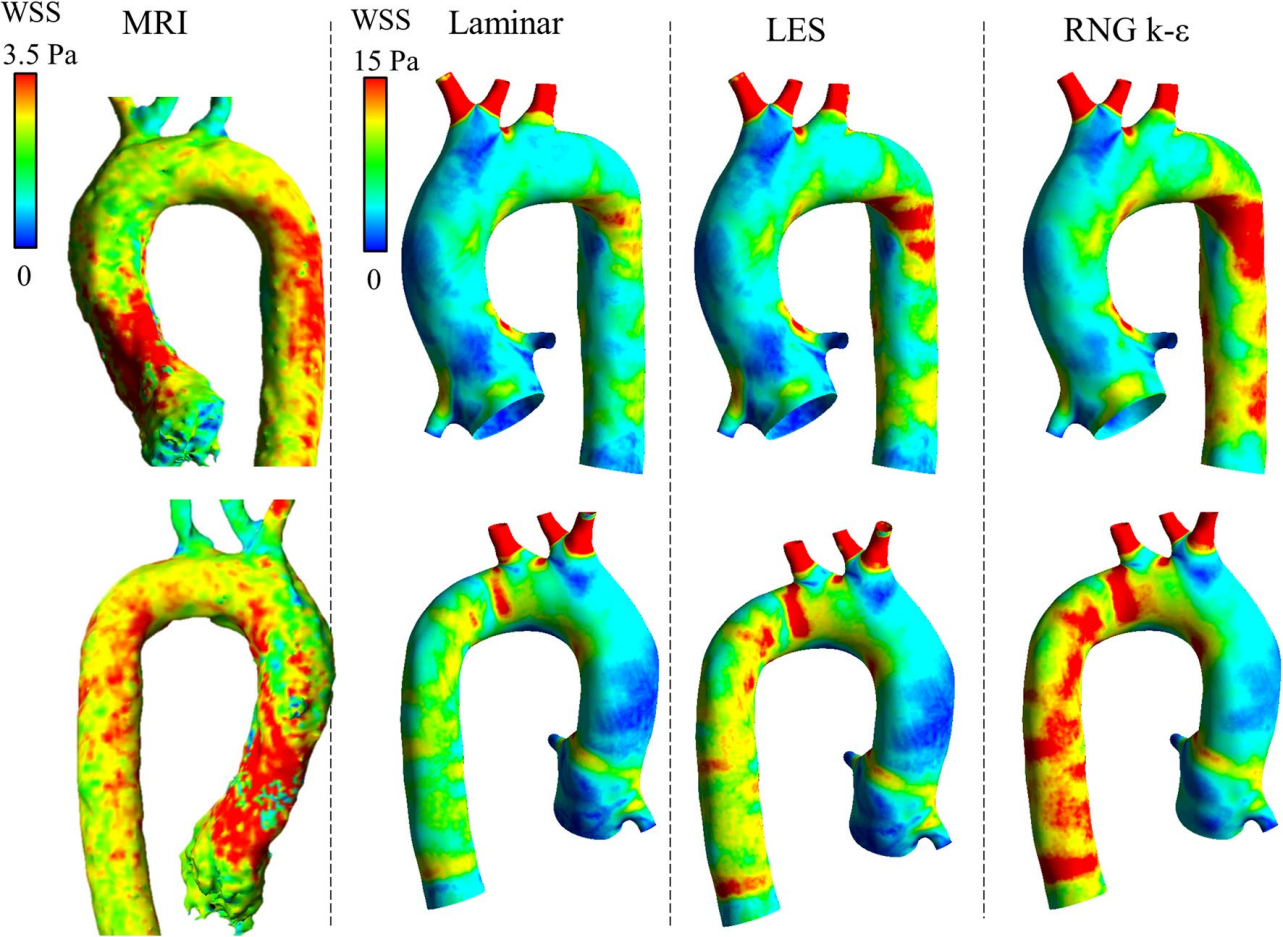
Distribution of WSS in the healthy adult. The WSS of the systolic peak are compared among 4D Flow MRI and three CFD computations using three turbulence models. The color scale of the MRI results was adjusted to conform to the area-averaged WSS of the CFD results. The average WSS was three to five times larger in CFD than in MRI

**Fig. 7:**
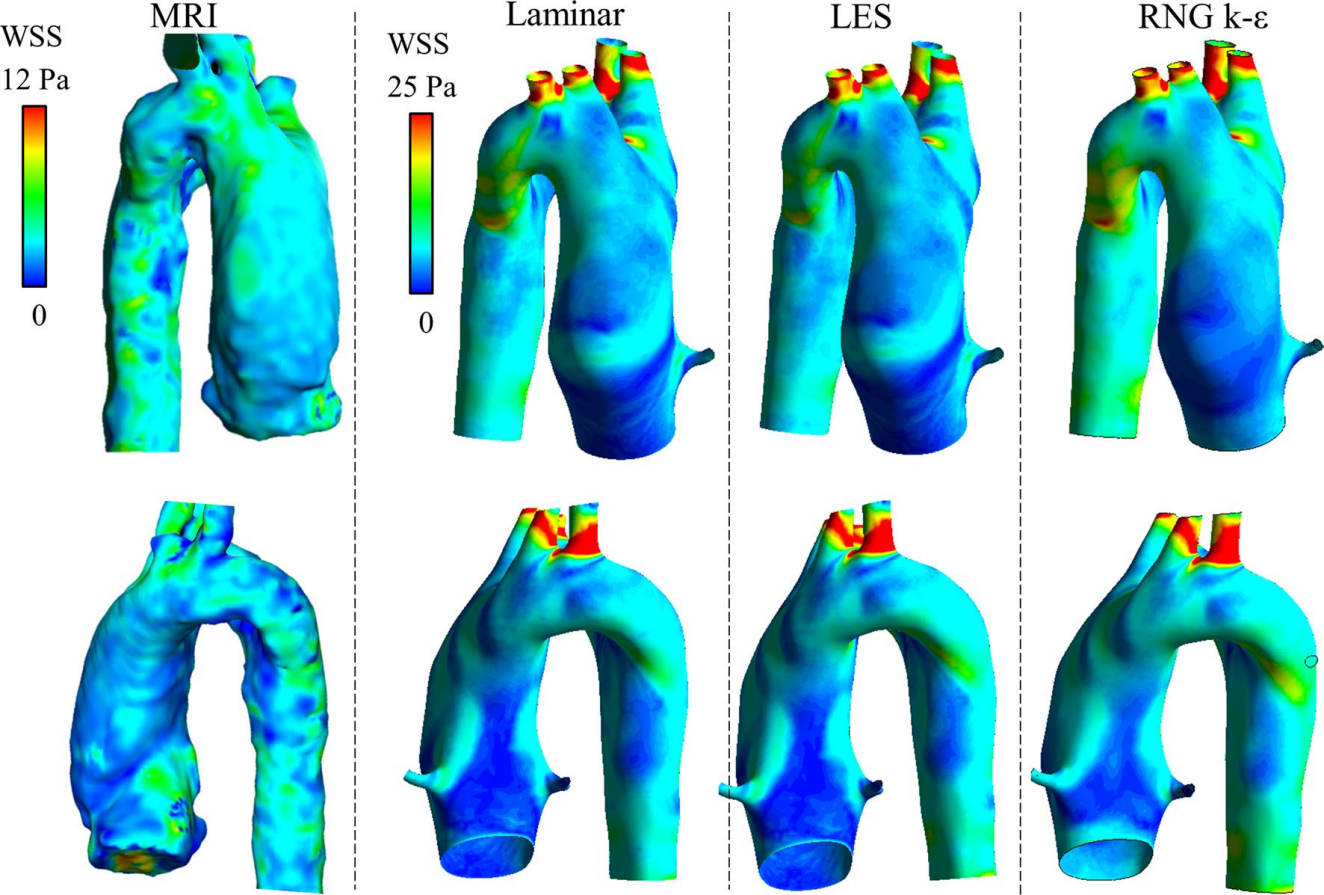
Distribution of WSS in the child with DAA. The WSS of the systolic peak are compared among 4D Flow MRI and three CFD computations using three turbulence models. The average WSS in the CFD and MRI was of the same order

**Fig. 8:**
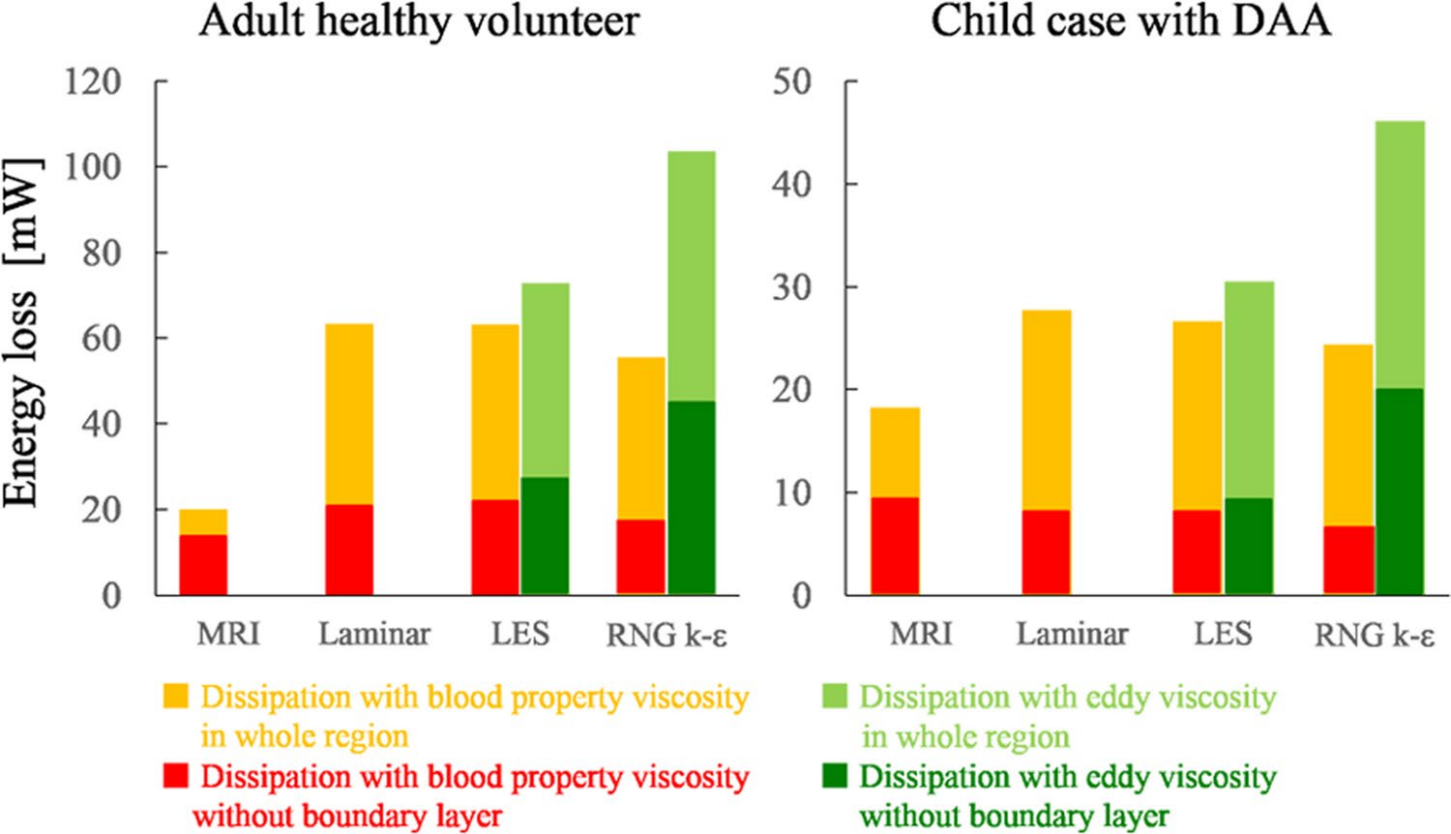
Energy loss. The EL of the whole aorta volume and that of the volume without a boundary layer are shown. In LES and RNG k–ε, which use eddy viscosity for turbulence computations, EL was calculated with the eddy viscosity and with the viscosity property of blood

The WSS distributions of the healthy adult and child with DAA are shown in Figs. 6 and 7, respectively. The average WSS in CFD was three to five times higher than that in MRI. The WSS in MRI was particularly lower in the neck vessels where the flow was much lower than that in CFD. However, the WSS distribution patterns were similar between CFD and MRI. The EL was also estimated to be larger in CFD than in MRI, as shown in Fig. 8. The EL in CFD was two to five times larger than that in MRI if the turbulent eddy viscosity was added; however, without the eddy viscosity and without the boundary layer, which is defined as the outermost voxel in MRI or the prism layers in CFD, the MRI and CFD EL values became similar.

## Discussion

The correlation of the velocity distributions between the 4D Flow MRI and CFD data was higher in the distal portion than in the proximal portion of the aortic arch. In the child, MRI detected helical flow in AAo, which was partly caused by the left ventricular twisting motion and partly by flow detachment around the aortic valve [13, 14]. In the present CFD method, a flat velocity profile was used on the top of the extruded boundary face as the inlet boundary condition, and the inlet velocity profile was developed in the linear extruded tube toward the axial through-plane direction without forming helical flow. Particularly, in the child, the correlation in AAo was worse because the helical flow, which is known to be affected by the age and size of the aortic valve [15], was stronger than that in the adult case. Goubegrits et al. have compared the CFD-simulated flow and MRI-measured flow in the aortic arch with coarctation; they reported that the helical flow in ascending aorta was not simulated accurately in CFD using the simplified plug velocity profile as the inlet boundary condition. The inflow velocity profile largely affects helical flow in the aorta, which largely affects WSS and pressure gradient in the coarctation of the aorta [16].

The distal flow was similar between the CFD and MRI results, and the correlation ratio was sufficiently high. The 3D curvature of the aortic arch develops helical secondary flow in the aorta [14], and the flow-velocity vector distribution mainly depended on the arch geometry rather than the inlet flow boundary condition. At the neck vessels, however, the flow velocity of MRI was much lower than that of CFD, particularly inside the divided arch of the child in the present study. Previous studies have indicated that small vessel diameters, low spatial resolution, and high velocity would cause an underestimation of the flow velocity [17], and decreasing vessel diameters led to larger discrepancies in the amount of flow between MRI and CFD.

Blood flow, which is normally thought to be laminar [18], can exhibit high frequency fluctuations, suggesting turbulent flow. According to the turbulent flow in a canine aorta observed with a hot film velocimeter, the presence of turbulence depended on the Reynolds number and Womersley number, which depend on the flow velocity [19]. The turbulence in the aortic arch has important implications for the pathophysiology of cardiovascular diseases. In the present study, the laminar scheme, RNG k–ε, and LES were examined for evaluating turbulence computations in the aortic arch. The RNG k–ε model was derived using a rigorous statistical technique and provided superior performance for flow involving rotation, boundary layers under strong adverse pressure gradients, separation, and recirculation [20]. In LES, large eddies are directly resolved, while small eddies, which are not dependent on the geometry, are calculated using a model. Large eddies contain most of the turbulent energy and are responsible for most of the momentum transfer and turbulent mixing. Therefore, LES offers more accurate flow than the k–ε model.

Stronger correlation with the MRI data was achieved by using a turbulent model, and the correlation was comparatively higher in the RNG k–ε model than LES. The classical theory of turbulence shows that the grid size required for computing the smallest size in the adult aorta is on the order of tens of micrometers, which is of the same order as leukocytes. In the actual computation of the aorta, the grid size was defined based on the EL and WSS calculations as tens of micrometers in the near-wall region and hundreds of micrometers in the center of the vessel. Thus, the grid size is too large to compute small eddies in the aorta, and the use of the laminar scheme resulted in a decreased correlation with the MRI results.

We evaluated EL with and without the boundary layer and with and without eddy viscosity in the LES and RNG k–ε models. Without the boundary layer, the EL value was the same among the MRI and CFD evaluations. On the other hand, in the boundary layer, the EL value was larger in the CFD. Most of the EL was caused in near-wall region where the velocity gradient is large and the MRI spatial resolution is insufficient, especially in the adult case with the lower spatial resolution. The EL underestimation in 4D Flow MRI has also been reported by Casas et al. [9]; they used simulated 4D Flow MRI data that were free from noise, which was underestimated by the low spatial resolution of MRI. Moreover, a rigid vessel wall assumption would cause overestimation of EL in CFD. The EL value was higher by performing the calculation with eddy viscosity. As mentioned above, the spatial resolution of the simulation in the center of the vessel is insufficient for depicting small eddies; thus, the calculations without eddy viscosity could underestimate the EL value.

The average WSS was three to five times larger in CFD than in MRI in the adult case, while the WSS in the CFD and MRI was of the same order in the child case with DAA. The same trends occurred in the EL evaluation, which shows that the spatial resolution in the healthy adult case was insufficient for evaluating the velocity gradient in the boundary layer. The WSS of MRI was underestimated especially in small vessels such as the neck branch where the flow velocity was underestimated. However, the distribution patterns of WSS were partially similar in PC MRI and CFD if the color scale was adjusted. In addition, the rigid wall boundary condition in CFD may cause an overestimation of the WSS because an elastic wall is known to reduce WSS [21–23]. The same trends were reported by Ooij et al. [24]; they measured wall shear stress in a cerebral aneurysm in vitro and in vivo with 4D Flow MRI and compared the results with CFD. The direction of the MRI WSS vectors was similar to that of CFD both in vitro and in vitro, and quantitative agreement between MRI and CFD was moderate due to the lower spatial resolution of MRI.

There were several limitations in the present study. First, MRI and CFD was performed only in two subjects. However, the design of present study is not a cohort study but an experimental one. Each subject has millions of sample points, which is sufficient to validate the flow in the aortic arch including flow separation and acceleration of the flow and vortex. The validation was performed experimentally in two subjects, but it would be preferred to perform validation in larger size in a future study. Second, we examined the aortic flow only in the peak systolic phase. Because our segmentation method was based on the velocity profile of PC images, we could not segment the volume of the diastolic phase when the flow velocity was imperceptible from PC imaging. Third, vessel wall was assumed to be rigid, which is reported to affect the wall shear stress [23]. Elastic parameters should be different throughout the aorta, and in the presence of atherosclerosis, these parameters drastically change. However, there is no current way to evaluate the distribution of the elastic property in vivo, and a uniform elastic parameter has been used in literature for calculating the whole aortic arch in FSI. Moreover, in this study, uniform rigid wall boundary conditions were used, which is one of the major limitations of this study. Thus, the flow is validated only in the systolic peak when the effect of the moving vessel wall was smaller and the flow velocity was easier to compare between MRI and CFD. In a future study, validation of the whole cardiac phase using accurate FSI with elastic parameter distributions should be performed. Fourth, the inlet boundary flow profiles may have been insufficient, and in future studies, they should be modeled by considering left ventricular contraction. Future studies should also include flow examinations over the whole cardiac phase in more complicated aortic diseases, such as a highly dilated aorta with aneurysm or an aortic valve stenosis with jet flow.

In conclusion, the flow velocity distributions were well correlated between MRI and CFD in the distal portion, but not in the proximal portion, because the helical flow caused by a twisting ventricle and the flow detachment around the aortic valve were not included in the CFD boundary conditions, whereas secondary flow is mainly formed by the curvature of the aortic arch. The velocity magnitude of MRI was underestimated in smaller vessels, such as neck branches or DAo. Among the three turbulence computations, the RNG k–ε model achieved the strongest correlation with MRI. The WSS estimate was lower in the 4D Flow MRI than in CFD because of the low spatial resolution and underestimation of the velocity magnitude, but the WSS distribution was similar. The EL value was also lower in MRI, and it is assumed that low spatial resolution caused underestimation of EL in the boundary layer.
